# Can Agro-Industrial By-Products Rich in Polyphenols be Advantageously Used in the Feeding and Nutrition of Dairy Small Ruminants?

**DOI:** 10.3390/ani10010131

**Published:** 2020-01-14

**Authors:** Fabio Correddu, Mondina Francesca Lunesu, Giovanna Buffa, Alberto Stanislao Atzori, Anna Nudda, Gianni Battacone, Giuseppe Pulina

**Affiliations:** Dipartimento di Agraria, Sezione di Scienze Zootecniche, University of Sassari, viale Italia, 39, 07100 Sassari, Italy; mflunesu@uniss.it (M.F.L.); gbuffa@uniss.it (G.B.); asatzori@uniss.it (A.S.A.); anudda@uniss.it (A.N.); battacon@uniss.it (G.B.); gpulina@uniss.it (G.P.)

**Keywords:** by-products, polyphenols, small ruminants, antioxidant, biohydrogenation, fatty acids, methane

## Abstract

**Simple Summary:**

In the Mediterranean area, where dairy sheep and goats are widespread, the use of by-products in the diet of small ruminants is an ancient practice. Today the great availability of industrial by-products produced at the local level (e.g., grape, olive, tomato and myrtle residues), appears to be a promising strategy for reducing competition with human edible foods and the cost of off-farm produced feeds since they are imported worldwide. Moreover, these co-feeds can contribute to reducing the ecological and water footprint associated with crop cultivation. The presence of bioactive compounds, such as polyphenols, confers added value to these materials. Several positive aspects are apparent when such by-products are included in the diets of small dairy ruminants, in particular on ruminal metabolism, animal health, and the quality of derived products.

**Abstract:**

Recently, the interest in industrial by-products produced at the local level in Mediterranean areas, resulting from fruit and vegetable processes, has increased because of their considerable amounts of bioactive compounds, including polyphenols. In this review, we analyze the most recent scientific results concerning the use of agro-industrial by-products, naturally rich in polyphenols (BPRP), in the diets of small dairy ruminants. Effects on milk production, milk and rumen liquor fatty acid profile, metabolic parameters, and methane production are reviewed. The feed intake and digestibility coefficients were generally depressed by BPRP, even though they were not always reflected in the milk yield. The main observed positive effects of BPRP were on quality of the milk’s FA profile, antioxidant activity in milk and blood, a reduction of rumen ammonia, and, consequently, a reduction of milk and blood urea. The expected beneficial effects of dietary polyphenols in small ruminants were not always observed because of their complex and variable matrices. However, owing to the large quantities of these products available at low prices, the use of BPRB in small ruminant nutrition offers a convenient solution to the valorization of residues arising from agricultural activities, reducing feed costs for farmers and conferring added value to dairy products at the local level, in a sustainable way.

## 1. Introduction

Presently, the reduction of global warming is a frequently debated problem. Each aspect of global warming relating to a reduction of the environmental impact arising from human activities has shown increasing interest.

Waste management represents a key element in strategies for reducing air and water pollution, greenhouse gas emissions, and health problems. One of the priority objectives indicated in the “7th Environment Action Programme of EU to 2020” regarding waste policy and managing waste is to maximize recycling and re-use [1].

The total amount of agro-industrial by-products in the European Union is around 16 million tons, with Germany (3 million of tons), the UK (2.6 million of tons), Italy (1.9 million of tons), France (1.8 million of tons), and Spain (1.6 million of tons) the top producers [2].

The livestock sector is considered an important player in global warming: the direct contribution of agriculture to total greenhouse gas (GHG) emissions is about 10% of all global emissions [3,4], 40% of which comes from enteric fermentation, with sheep and goats accounting for about 7% and 5% of the global enteric emissions, respectively [4]. An additional environmental impact of the livestock sector is ascribable to feed production. Growing and processing, transport and land use, and changes in land use are the main global sources of GHG emissions in animal feed production.

In this scenario, the use of agro-industrial by-products as feed ingredients could represent an important component of the global strategy to reduce the environmental impact of both agro-industrial and livestock production.

The use of some by-products as animal feed has been explored and represents one of the easiest ways to exploit these materials [5]. By-products such as beet pulp (resulting from the sugar manufacturing process), corn gluten feed (resulting from the extraction processes of the starch), gluten and germ from corn, and soybean hulls (mainly consisting of the outer covering of soybean), soybean meal, linseed meal, corn gluten meal, cottonseed meal, and sunflower meal (obtained by grinding the material resulting after oil extraction), are commonly used in the animal feed industry, owing to their high nutritional values, related to their significant amounts of fiber and/or protein, depending on the feed.

Not only these “traditional” by-products but also those derived from fruit and vegetable processes are the objectives of the study [6]; these by-products seem to have applications in animal nutrition because of their considerable amounts of bioactive components [7], especially polyphenols, such as proanthocyanidins (tannins), or flavonoids [8]. These compounds, included at low or moderate levels in the diets of animals, have positive effects on productive performance and health [9,10]. In addition, the transfer of these natural antioxidants in animal tissues has improved the quality of livestock products, which is related to their ability to increase oxidative stability [11].

Considering their composition, these by-products can be defined as agro–industrial by-products naturally rich in polyphenols (BPRP). When included in a ruminant diet, BPRP can lead to several advantages: serving as an alternative to the disposal of these products, reducing the feeding cost for farmers, and conferring added value to dairy products (in terms of improving the quality and sustainability of their production).

Recently, great attention has been paid to the health benefits that livestock, humans, and the environment can achieve when livestock are able to forage on a phytochemically rich landscape [12]; these advantages are related to plant diversity and the large variety of phytochemicals, including polyphenols.

As BPRP are a great source of phytochemicals, their use may represent a useful way to bring the typical diets of ruminants closer to healthy foraging on phytochemically rich landscapes, instead of foraging on simple mixtures or monoculture pastures or consuming high-grain rations in feedlots.

The main limitations to the wide use of BPRP in livestock, represented by their high variability in the composition of nutrients [13], could instead constitute an advantage for the valorization of these biomasses as a feed. Moreover, the local and seasonal availability of some BPRP represents a limitation to their wide use, as the production of fruit and vegetable residues is often seasonal, and in many cases, BPRP are produced by small or medium size implants, resulting in low availability [14].

This review summarizes the available literature on the use of agro-industrial by-products naturally rich in polyphenols in the feeding and nutrition of dairy small ruminants. The effects on animals’ performance, milk production and composition, and milk quality are examined. In addition, the effects on ruminal metabolism, metabolic parameters, methane production, and associated environmental impacts are reviewed.

## 2. By-Products Naturally Rich in Polyphenols

The utilization of agro-industrial by-products as a source for high value-added products represents one of the methods for the valorization of this biomass. The positive aspects related to the use of bioactive compounds (e.g., polyphenols) in ruminant nutrition [9,10] have increased interest in using by-products rich in polyphenols as dietary ingredients in ruminant feeds [13,15].

### 2.1. Chemical Composition of Agro-Industrial By-Products Naturally Rich in Polyphenols

Table 1 presents some BPRP that have been studied as ingredients in the diets of dairy small ruminants. The chemical compositions of these materials are largely variable; for example, the NDF content ranges from about 100 g/kg of DM for apple by-products to 600 g/kg of DM and more, which was reported for exhausted myrtle berries, olive cake, and pomegranate seeds. Concerning the CP content, large values can be observed for all by-products arising from the tomato industry, ranging from 157 to 217 g/kg of DM. Wide variability could be also observed among different by-products from the same source. For example, the winery industry produces different residues: grape pomace, grape pulp, grape seeds, and grape stalk. Moreover, differences among grape by-products can also be due to the cultivar, stage of ripening, and agro-climatic conditions. The exhausted myrtle berries, collected in two different liquor factories, varied in lipid content from 54 to 110 g/kg of DM [16,17].

### 2.2. Phenolic Compounds of Some Agro-Industrial By-Products Naturally Rich in Polyphenols

**Figure 1:**
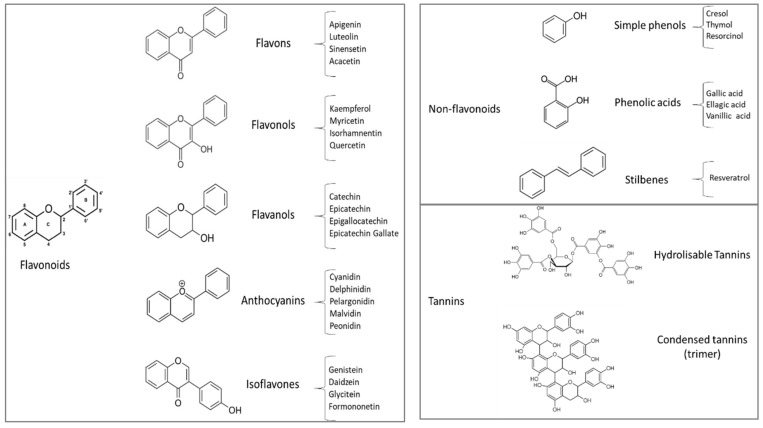
Main classes of polyphenols: flavonoids, non-flavonoids, and tannins.

Polyphenols are products of the secondary metabolism of plants. The synthesis of these compounds derives mainly from shikimate and the acetate pathways during the normal development of a plant, or under different stress conditions [42]. Although not completely defined, the biological role of polyphenols seems to be related to some plant defense mechanisms against pathogens, herbivorous, insects (antibiotic and anti-feeding actions), and solar radiation [43]. More than 8000 different structures have been identified, including simple molecules, such as phenolic acids, or more complex structure, such as tannins. Polyphenols are characterized by at least one aromatic ring having one or more hydroxyl groups and can be classified as different classes of compounds, according to their chemical structures: flavonoids, non-flavonoid, and tannins (Figure 1).

Flavonoids constitute the most important single group, with more than 5000 described compounds [44]. Their chemical structures consist of two aromatic rings linked through three carbons that usually form an oxygenated heterocycle (Figure 1). This class of flavonoids includes several subgroups, such as flavones (e.g., apigenin), flavonols (e.g., quercetin, myricetin), flavanones (e.g., naringenin, hesperidin) isoflavones, and anthocyanidins [44].

Among non-flavonoids, the most common structures are represented by simple phenols (e.g., cresol, thymol, and resorcinol), phenolic acids (e.g., gallic, vanillic, and syringic), and stilbenes. Phenols and phenolic acids can be found either free or in their corresponding methyl, ethyl ester, and glycoside forms.

Tannins are typically divided into two groups, hydrolysable and condensed tannins. Hydrolysable tannins (HT) chemically consist of a carbohydrate (mainly glucose) whose hydroxyl groups are esterified with phenolic acids (gallic acids or hexahydroxydiphenic acid). Condensed tannins (CT) are polymers of the flavan-3-ol (dimers, trimers, tetramers, but also very high polymerized structures) and are also known as proanthocianidins. These highly hydroxylated molecules can form insoluble complexes with carbohydrates and proteins. These compounds can confer astringency to foods because of the precipitation of salivary proteins. This is an important aspect in ruminant feeding and nutrition because a high amount of these compounds in plant feed can lead to a reduction in voluntary feed intake and nutrient digestibility with a negative impact on animal performances [45]; for this reason, tannins and polyphenols in general have been historically known as antinutritional factors.

Several BPRP used in small ruminant feeding and nutrition contain polyphenols (Table 2); the number of polyphenols varies based on the processing of the original materials. For example, myrtle berries (*Myrtus communis* L.) are very rich in anthocyanins [46,47], which have been detected at high amount in hydroalcoholic extracts and are the basis of production of the commercial liqueur (Mirto) [48], but they were not detected in exhausted myrtle berries [49]. On the other hand, a hydroalcoholic solution showed moderate levels of phenolic acids (gallic and ellagic acids), which were the most representative compounds in the exhausted myrtle berries.

The biological activities of polyphenols have been largely investigated in humans and evidence antioxidant abilities [70] and positive consequences on health, such as cardioprotective [71,72], anti-inflammatory [73], antidiabetic [74], and anticancerogenic effects [75,76]. These natural compounds have also been designated as important leads for multi-target drug development [77].

The effects of polyphenols in the diet on rumen digestion and post-absorption [10,78] have been reviewed. Despite the recognized reduction of voluntary feed intake in animals fed high amounts of tannin rich plants, it has been observed that moderate levels of polyphenols in the diets of ruminants can improve the performance of animals owing to a better utilization of dietary protein [45]. Indeed, polyphenols are able to bind dietary proteins, thus reducing their ruminal degradation and leading, in turn, to an increase in amino acid flow to the small intestine [79,80].

The use of feed rich in polyphenols in ruminant nutrition can also improve the quality of the derived foods. The ability of polyphenols to modulate the rumen biohydrogenation of polyunsaturated fatty acids (PUFA) [10] leads to an improvement in the quality of the lipid fraction of dairy products [81], by increasing the concentration of beneficial fatty acids (e.g., PUFA, vaccenic, and rumenic acids), reducing the ruminal biosynthesis of skatole, and increasing the oxidative stability of products [8].

The positive effects of polyphenols on animals’ health have been evidenced by the reduction of intestinal parasites in sheep [82,83], improvements in the cell-mediated immune response [84], a reduction in inflammatory processes [85], and improvements in the antioxidant status of animals [86,87].

Of course, the cost of traditional feedstuff, its safety for animals, and the attractiveness of alternative uses influence the choice of by-product utilization [6].

## 3. By-Products Naturally Rich in Polyphenols in Small Ruminant Feeding and Nutrition

There is a significant amount of literature on the role of dietary polyphenols (mainly condensed tannins) in ruminant feeding and nutrition. However, clarifying the contribution of each BPRP on intake and animal performance, considering the attribution of a specific effect to its polyphenol, is quite difficult and risky, because these materials are often characterized by complex chemical compositions. In addition, several works report only the total polyphenolic content or the main classes of polyphenols, omitting their complete profiles. Furthermore, because of the different effects that these compounds can have on the animals, the certain attribution of their effects is quite difficult.

### 3.1. Effect on Voluntary Feed Intake 

**Figure 2:**
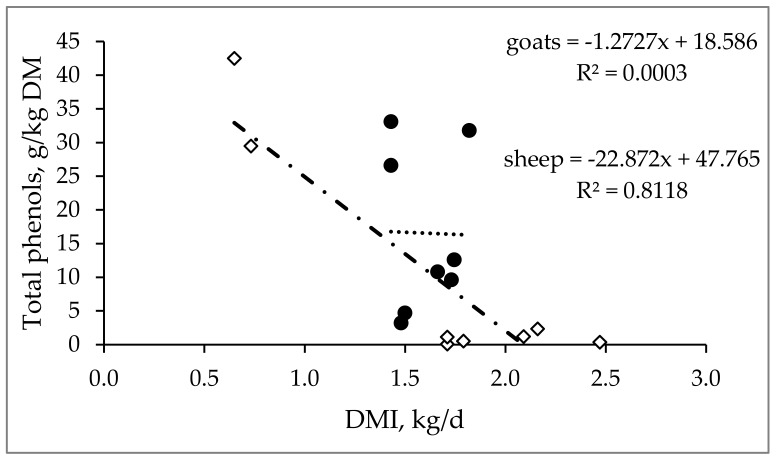
Relationship between total phenol content of by-products naturally rich in polyphenols (BPRP) (expressed in g/kg DM) and dry matter intake (DMI, expressed in kg/d) in sheep (◊) and goats (●) (goats: [40,93,94,107]; sheep: [16,17,90,91]).

The inclusion of BPRP in the diets of small ruminant seems to decrease voluntary feed intake in sheep but not in goats. In particular, a negative relationship has been observed between the amount of total phenols contained in BPRP (expressed in g/kg DM) and DMI (expressed in kg/d) only in sheep (y = 22.872x + 47.765; R^2^ = 0.8118) [16,88,89,90,91], as reported in Figure 2. In contrast, in goats, this association was not observed (y = −1.2727x + 18.586 R^2^ = 0.0003; Figure 2) [40,92,93,94]. Probably, considering their different feeding behaviors (goats are intermediate feeders and ewes are grazers) [95], goats developed more strategies against these types of feeds rich in polyphenols (e.g., the presence of proline-rich proteins in the saliva [96] and a higher capacity of the saliva to bind tannins [97,98,99,100], which can help this species better control the toxicity of tannins than grazers [101]. However, the different behaviors between species do not appear when the levels of polyphenols in their diets are moderate [96]. Regarding possible differences between the two species, goats, compared to sheep, show the best ability to use BPRP. However, by adopting some managerial practices to inactivate tannin content (e.g., the use of wood ash, urea, PEG, or ensiling) [78], tannins can be advantageously used in sheep nutrition, as well. In addition, for both species, especially when the animals are reared in an extensive system, special attention should be paid to the use of BPRP. In fact, grassland and shrubland, especially those typical of the Mediterranean area, are naturally rich in polyphenols, and, even though they show seasonal variation in their chemical compositions [102], simultaneous utilization with BPRP could lead to an excessive daily amount of dietary polyphenols. Similarly, some forages (e.g., *Vicia sativa* L., *Lotus corniculatus* L., *Hedysarum coronarium* L., and *Lotus pedunculatus*) can also contain a high number of polyphenols that affect the performance and metabolism of animals [103,104,105,106].

### 3.2. Effect on Digestibility

The introduction of BPRP in the diets of small ruminants usually depresses nutrient digestibility [22] compared to traditional feedstuffs (e.g., concentrates and forages). 

In terms of CP digestibility, the use of BPRP decreases the digestibility of proteins [22,93], likely because of the ability of tannins to bind proteins [22]. The same results are evidenced for NDF digestibility with a supplementation of BPRP [22], likely because of the formation of an indigestible complex between tannins and the cell wall carbohydrates in the rumen.

Generally, the lower digestibility coefficient that exhibits BPRP is linked to a high level of lignin and tannin content [108] and to the industrial process to which the by-products have been subjected. In fact, the heating of the material necessary to extract oil, wine, and tomato during the industrial process increases the amount of N linked to the cell wall or that of the tannin complex in the residuals (by-products) as a result of the Maillard reaction [109], which reduces CP digestibility. In this sense, the use of PEG can help increase the CP digestibility of by-products [22].

In some cases, considering their high NDF and ADF content, which limits the digestibility, some by-products (e.g., tomato pomace) are comparable to low quality forages [110].

Compared to sheep, goats seem to have a better ability to digest BPRP [109,111], especially when their polyphenolic profile is mostly represented by condensed tannins [112]. The different behaviors in BPRP digestibility between sheep and goats could be linked to divergences in their tannin activity response [113], especially in the degradation of tannin–protein complexes [114] and in the ruminal microbial population [89].

### 3.3. Effect on Blood Metabolites

In sheep and goats, literature on the effects of BPRP on metabolic parameters is quite consistent and concerns, independent for each considered species, especially blood urea decrease [40,93,108,115] are probably associated with the ability of tannins to bind dietary proteins, thereby reducing their degradability at the rumen level, whereas others haemato-parameters are not affected [17,90,91].

The positive effects of polyphenols on oxidative status were detected both in goats [115] and ewes [24]. The antioxidant effects, in vivo, are rather complex. In fact, polyphenols can exert direct antioxidant activity as a consequence of their absorption along the gastrointestinal tract and because of their deposition in the tissues [116,117]. Other authors suggest an indirect mechanism. Considering that dietary polyphenols are poorly absorbed in the intestine [85], in particular in ruminant species, their effects could be mediated by chelating pro-oxidant metals at the intestine level with a reduction of lipid peroxide production [118,119]. 

Furthermore, the widespread idea is that the use of by-product additives in animal nutrition does not negatively influence animal welfare and that additives, especially additives rich in tannins, can exert an anthelmintic effect in both sheep and in goats [120]. Plants, thanks to their high variety of bioactive substances, have demonstrated an important medicinal potential for controlling gastrointestinal parasites in ruminants [121]. Phytogenic feed additives (plant derived products) are used as animal feed; particularly in swine and poultry, these additives have received increasing attention as they can, to a certain extent, obviate the use of antibiotics [122]. In this sense, the inclusion of BPRP in the diets of ruminants could be considered a small step in the right direction to reduce the use of antibiotics. 

### 3.4. Effect on Rumen Parameters

The effects of dietary polyphenols on volatile fatty acids (VFA), ammonia, and methane (CH_4_) production have been widely investigated. Polyphenols have evidenced the capacity to reduce urea and CH_4_ emissions, thereby decreasing the environmental impact of small ruminant species, as observed in our previous work [123], where BPRP was advantageously used to improve nitrogen balance in ewes.

As reviewed by Vasta et al. [10], the decrease in CH_4_ production could be due to a direct or indirect consequence of using tannins, in particular, because of an interaction of by-products with ruminal microorganisms or because of an inhibition of fiber digestion. In other words, the decrease in CH_4_ production first is due to the interactions between secondary metabolites and ruminal microorganisms and second due to a decrease in hydrogen ions because of the lower feed degradability [124]. However, unfortunately, anti-methanogenic activity is often accompanied by a reduction in organic matter (OM) digestibility and thus in animal productivity [125].

In Table 3, the main effects on the ruminal parameters of the dietary inclusion of different BPRP in sheep and goats are reported. One of the most frequent effects is the reduction of the total concentration of VFA, which is associated with a reduction in microbial activity as a consequence of lower feed degradability [125]. Among individual VFA, acetate is often reduced. This can be ascribed to the inhibiting effects of polyphenols (tannins in particular) on the activities of cellulolytic bacteria, whose main product is acetate. On the other hand, in some cases, an increase in propionic acid concentration is reported, which, in turn, leads to a decrease in the acetate to propionate ratio. This is important from an environmental point of view, considering that a negative correlation exists between the production of CH_4_ and that of propionate because of their competition for hydrogen. It should be noted that the anti-methanogenic activity of polyphenols is also related to their effect on methanogens [126].

Another important aspect is represented by the reduction of ammonia, which has been reported by several authors. Considering that rumen ammonia is generated from protein degradation, its reduction is probably associated with a decrease in protein degradability [129]. Indeed, the capacity of polyphenols to bind dietary proteins and to reduce the extent of their ruminal fermentation is well-known [79,80]. This last aspect is important for two reasons: the improvement of nitrogen utilization by animals, from a nutritional point of view, and the reduction of nitrogen excretion from an environmental prospective. For both species, except for the study in sheep by Correddu et al. [123], the influence of BPRP on rumen parameters seems to become stronger as the dose of polyphenols in the diet increases. However, not only the dose but also the type of BPRP and the high variability in the composition of the nutrients [13] must be taken into account.

### 3.5. Effect on Milk Production and Composition

The effects of BPRP supplementation in small ruminants’ diets on milk production and composition did not yield univocal results (Table 4). In general, the inclusion of BPRP in the diets of sheep and goat showed weak effects on milk yield and composition.

**Figure 3:**
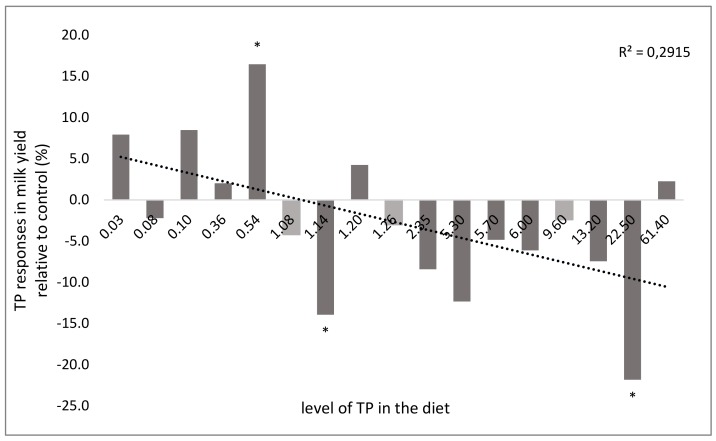
Effect of total polyphenol (TP) concentration in the diet on sheep (dark grey) and goat (light grey) milk yield calculated as a percentage of the increase or decrease compared to the control group; * indicates a significant difference (*p* < 0.05) compared with the control group.

A positive effect on milk yield was obtained when grape pomace was included in the diet of sheep [17]. Other works, where vinery by-products were used [26,28,91], did not show a variation of the milk yield compared to unsupplemented animals. No effects [16] or depressive effects [17] of exhausted myrtle berries (EMB) on milk yield have been observed among Sarda dairy sheep. The depressive effects observed in the last trial seem not to be related to the level of exhausted myrtle berries polyphenols in the diet, since this level was similar to that used in the previous experiment (28 vs. 22.6 and 44.3 g/kg of DM); the authors explained this effect by the reduction of DMI because of a high content of aNDFom and of hydrolysable tannins in the BPRP. Such differences in the results could also arise from different interactions between exhausted myrtle berry polyphenols and the other ingredients of basal diets, as suggested by Toral et al. [81]. Negative effect on milk yield were also observed by the inclusion of olive leaves in the diet of sheep [32]. Even if a univocal effect of BPRP on milk yield of sheep and goats is not easy inferable, a tendency can be observed when the milk yield (expressed as a percentage difference between the control and treatment groups) is reported as a function of the total phenol concentration in the diets (Figure 3). Indeed, Figure 3 shows that a positive response in the milk yield is obtained when polyphenols are present at low concentrations in the diet, whereas, by increasing the polyphenol concentration, a general depressive effect can be observed. Although this relationship is not strong (R^2^ = 0.2915, Figure 3), it is possible to deduce that increasing the levels of BPRP in the diet will decrease milk production in both sheep and in goats. Thus, in terms of the effect of BPRP on milk yield, the two species appear to respond in the same manner.

Considering the effect of BPRP on milk composition, the literature reports contrasting results. In fact, a positive, negative or lack of effect for the same BPRP can be observed (Table 3) in the example of grape or tomato residues. Regarding the differences between the two ruminant species (Table 4), an increase of milk fat content in goats can be observed, whereas no variation or a reduction of milk fat content can be observed in studies on sheep. Most of the literature, however, reports no effect of protein concentration on BPRP. Negative effects were reported by Nudda et al. [17] when a dairy sheep diet was supplemented with tomato and grape by-products. The depressive effect of tomato by-products on milk protein concentration was previously reported by other authors [132]. The decline in milk protein content was explained by the reduced dietary energy supply [132] or by the lower rumen degradability of the tomato by-product [17].

In general, there is a lack of information regarding milk urea concentration in response to BPRP inclusion in small ruminant diets. The important effects of dietary polyphenols on the rumen degradability of proteins, reducing milk urea concentration, have been previously reported [79,80]. Similar results are also expected after the inclusion of BPRP in ruminant diets, as evidenced in the [16], suggesting the potential role of polyphenols in ruminant nutrition to improve nitrogen utilization and reduce nitrogen excretion in the environment.

### 3.6. Effect on Mik and Cheese Fatty Acid Profile

Considering the link between diet and health, great attention is presently placed on the quality of foods. Consumer choice, in particularly in developed countries, is directed toward foods that are not harmful, which can preferably promote health. Excluding the presence of exogenous compounds (e.g., toxic xenobiotics), the quality of foods mirrors the quality of their constituents. The quality of animal-derived foods is strongly associated with the characteristics of their lipid fractions. The typical high content of saturated FA in animal fat has recently been upgraded by a cohort study [133], showing that a higher saturated fat intake is associated with a lower risk of stroke. Ruminant fat contains PUFA belonging to the omega 3 and omega 6 families, as well as conjugated linoleic acid (CLA) isomers that are considered beneficial for health. In particular, rumenic acid, the *cis*-9,*trans*-11 conjugated isomer of linoleic acid, has demonstrated healthy effects, such as antiatherosclerosis, anticancer, antidiabetic, and anti-inflammatory activities in laboratory animals [134] and anticholesterolemic and anti-atherosclerosis effects in humans [135,136].

The lipid content of ruminant-derived foods, in particular their FA composition, is largely influenced by the activity and metabolism of rumen microflora [137]. The inclusion of polyphenols in animal diets can modulate rumen microorganism activities [10]. Thus, studies have been carried out to research the exploitability of modulating rumen microbiota, using dietary polyphenols to improve the FA profile of foods [8,80] and increase their nutraceutical FA content (e.g., PUFA and CLA). BPRP, as sources of exploitable polyphenols, can be used with the same goal.

The inclusion of pomegranate pulp (648 g of dried pomegranate pulp in each kg of DM of the diet) in the diet of sheep was effective in reducing the concentration of SFA and increasing that of PUFA [37]. Among individual FA, the authors found a reduction of myristic (C14:0) and palmitic (C16:0) acids and an increase of vaccenic (C18:1 *trans*-11), rumenic, and alpha-linolenic acids (C18:3 *n*-3). The use of pomegranate seed pulp (120g/kg DM; 4.7 g of total phenol on kg DM of diet) gave similar results in two experiments on goats (different breeds), higher concentrations of PUFA and CLA and among individual FA, higher concentration of vaccenic, rumenic, and alpha-linolenic acids [40,130]. A reduction of SFA and an increase of unsaturated fatty acids (UFA), both mono and polyunsaturated, in particular vaccenic acid (2.02 vs. 1.16 g/100 g, treated vs. control), were also observed in the milk of dairy goats via the partial substitution of alfalfa hay with pistachio by-products [93]. The addition of PEG, to minimize the effect of tannins, did not change the milk concentration of vaccenic acid compared to the control [93], thereby confirming the ability of tannins to reduce the last step of the rumen biohydrogenation of FA, as previously observed in vitro by several authors [138,139]. Another study [40], with similar dietary treatments, confirmed the suitability of pistachio by-products to increase the milk concentration of vaccenic acids, even though, contrary to the first work, the total concentrations of SFA, UFA, and PUFA did not change.

Positive effects on the quality of milk FA profile were documented also by using by-products arising from the processing of *Rosmarinus officinalis*, olive, tomato, and lentil. In particular, distilled *Rosmarinus officinalis* spp. leaves in the diets of goats (10 and 20%) reduced the milk concentration of C14:0 and increased PUFA, particularly linoleic acid (C18:2 *n*-6) [131]. The inclusion of olive cake or tomato pomace at a level of 30% DM of the diet of Awassi ewes increased oleic acid (18:1 *cis*-9) content, whereas olive leaves or lentil straw (at the same level of inclusion) increased PUFA *n*-3 and rumenic acid contents [32].

The inclusion of vinery by-products in the diets of dairy sheep did not produce univocal results. The supplementation of dairy ewes’ diets with different doses of grape pomace (5 and 10% of dietary DM) did not affect the milk’s FA composition [26], whereas the use of grape seeds (about 12% of dietary DM) was effective in decreasing SFA and increasing monounsaturated fatty acid (MUFA), PUFA, and CLA [140], which resulted in a reduction of the atherogenic index (AI), the thrombogenic index (TI), and an increase in the hypocholesterolemic to hypercholesterolemic ratio (h:H). The lack of effects on the FA profile observed by Manso et al. [26] was ascribed to the low levels of inclusion used in the experiment compared to other studies on dairy ewes and cows, where significant effects were observed [21,141]. However, considering the significant results also achieved in experiments using moderate levels of BPRP [40,130,140], some considerations may be formulated. In the experiment of Manso et al. [26], the diets included 2.7% (on DM basis) of linseed oil. The role of vegetable oils in altering the FA composition of ruminant products has been deeply investigated [142,143] and represents one of the most commonly used strategies to improve the nutritional quality of milk fat [144]. The presence of linseed oil in the experimental diets, including that of the control group, could have masked the possible effects of other dietary ingredients (i.e., grape pomace polyphenols). This hypothesis is supported by the results obtained in our recent study [59] on sheep fed exhausted berries of myrtle. Similar to Manso et al. [26], sheep, including those belonging to the control group, were fed a diet with a lipid source (extruded linseed), and no effect on the milk FA profile was observed, confirming that the effects of polyphenols are complicated by the complex interactions between different factors, including the other ingredients of the diet [81]. Overall, the effect of BPRP on the milk fatty acid profile seems to be similar between sheep and goats and leads to a decrease in the SFA content and to an increase of PUFA in milk.

The modulation of ruminal biohydrogenation by dietary polyphenols can increase the amount of total *trans*-FA in the milk, mainly vaccenic acid and other isomers of *trans*-C18:1, that may potentially be undesirable (e.g., C18:1 *trans*-9 and elaidic acid).

Moreover, the lipid content and FA composition of BPRP could also affect the milk FA composition. Some by-products, in fact, contain an interesting lipid fraction (Table 1), varying (on a DM basis) from 10% in grape seeds, tomato pomace, and exhausted myrtle berries, to 22 and 28% in olive cakes and winery sediment, respectively. The level of inclusion of a considered BPRP in the diet and the fatty acid profile of its lipid fraction should be considered important factors that can influence the fatty acid composition of animal products. For example, the inclusion of 300 g/d (about 12% DM of the diet) per head of grape seed (10% of oil) in a sheep diet largely affected the milk fatty acid profile [140]. This result is related to the specific fatty acid composition of the by-products lipidic fraction (more than 70% was represented by C18:2 *n*-6, linoleic acid) and to the high level of inclusion of this by-product (and, in turn, of its lipidic fraction) in the diet of sheep. Another example is given in the work of Abbeddou et al. [32]. Here, the authors evaluated the effects of different BPRP (characterized by different lipid fractions and fatty acid profiles), included at the same level in the diet of sheep, on the milk FA profile. The results evidenced a large variability of the effects, depending on the specific fatty acid profile of the considered BPRP and on the level of its lipid fraction. However, the effects of polyphenols on biohydrogenation should not be neglected, as evidenced, for example, by the high levels of C18:1 *trans* isomers found in the milk of sheep fed BPRP with higher levels of total polyphenols.

The inclusion of 300 g/d (about 12% DM of the diet) per head of grape seed (10% of oil) in the sheep diet [140] markedly increased the C18:2 *n*-6 content in milk fat, with this FA being the most abundant in these by-products (more than 70% of the total FA). Similar results have been reported by Abbeddou et al. [32] by using different BPRP characterized by different lipid fractions and fatty acid profiles. However, the effects of polyphenols on biohydrogenation should not be neglected, as evidenced by the high levels of C18:1 *trans* isomers found in the milk of sheep fed BPRP with higher levels of total polyphenols.

Another important feature is the potential transfer of antioxidant compounds (or relative metabolites) from BRPR to milk and dairy products [116,117], with advantageous effects on the physicochemical and sensory properties of milk and derived dairy products—protection, to a certain extent, from oxidative processes, with a consequent reduction of undesirable product formation (off-flavors and potentially toxic compounds) arising from the oxidation of lipids and proteins. The latter aspect is very important since antioxidant compounds can contribute to extending the shelf-life of products, as oxidative reactions are an important process that contributes to the deterioration of foods characterized by highly unsaturated lipids, which are extremely susceptible to oxidation. Positive effects, in terms of increased milk antioxidant capacity, are reported for both sheep and goats when BPRP are included in their diets (grape residue flour was added to the diets of dairy sheep) [28,94]. In these studies, a direct antioxidant effect could be hypothesized even though the antioxidant outcome of the polyphenols could be also ascribed to an indirect mechanism mediated by its effect on the general oxidative status of the animal [87].

When antioxidant activities are studied, special attention should be given to the qualitative and quantitative aspects of FA in the considered foods. Sometime the antioxidant effect of dietary BPRP can be masked by a high content of PUFA in milk, which are highly susceptible to oxidation. For example, Valenti et al. [37] found a lower antioxidant capacity in the milk of ewes fed pomegranate pulp (61.4 g/kg DM total phenols, mainly tannins) compared to the control group, likely because of the higher peroxidable FA in the first group. A similar finding was observed by Correddu et al. [145] in a diet supplemented with grape seed by-product. In that study, under exposure to light, the milk of animals with the higher UFA concentrations tended to have higher accumulations of lipid hydroperoxides, but when expressed as a ratio between the oxidation product and UFA, the milk of animals fed grape seeds demonstrated a higher antioxidant capacity. Studying the effects of different BPRP on milk FA composition and antioxidant status in the diets of ewes, Abbeddou et al. [32] found that the diet with the highest content of total phenols (olive leaves) led to the lowest antiradical activity of the milk. Consequently, the milk fat of the animals fed this PBRP was also the highest, with 18:3 *n*-3 and total PUFA; thus, part of the antiradical compounds might have been immediately spent to counteract oxidation.

## 4. Systemic Perspective of Using By-Products Rich in Polyphenols in Ruminant Nutrition

**Figure 4:**
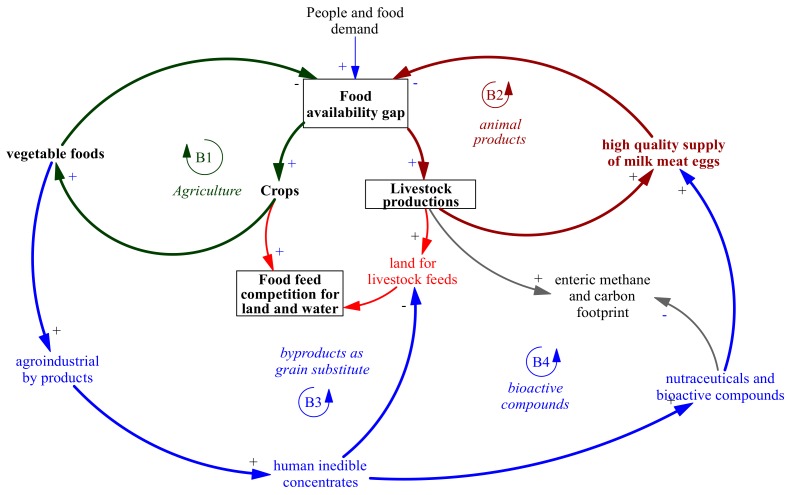
Causal loop diagram of the beneficial role of the use of agro-industrial byproducts in the food chain. Arrows indicates causality, whereas polarity signs, + and -, indicate positive and negative correlation, respectively. B indicates balancing system loops.

A general picture of the nutritional and environmental roles of by-product use in animal nutrition can be summarized from a systemic perspective that focuses on by-products in agricultural and food-based contexts. System thinking and analyses are often used to qualitatively analyze the interactions among system elements using causal maps or causal loop diagrams. These methods allow one to increase the complexity of understanding by describing the system and highlighting the feedback loops that connect the most important variables and elements in order to determine possible future behaviors and effects. Causal diagrams have already been applied in agriculture and food systems to highlight sustainable links and suggest alternative policies [146,147,148]. A big picture of the by-products in agricultural and livestock systems is summarized using a causal loop diagram in Figure 4. This diagram was developed using the conventional annotation adopted to build a causal loop diagram via a system dynamics technique [149]. Arrows indicates causality, whereas the polarity signs, + and −, indicate positive and negative correlations, respectively. R and B indicate reinforcing and balancing system loops, which, over time, drive exponential growth (unsustainable) or the asymptotic behavior of the system (sustainable) [149].

The beneficial role of the use of agro-industrial by-products in the food chain is evidenced in Figure 4 by following the arrowhead links describing the system structure.

People drive food demand. When food demand increases over time, food availability gaps also increases. An increase in food demand will stimulate the production of cultivated crops and livestock. On the one hand, agriculture will enhance vegetable production to provide grain, fruits, and vegetables (the green arrows and balancing loop of agriculture are show in B1 in Figure 4). On the other hand, livestock will provide milk, eggs, and meat (the brown arrows and balancing loop of animal products are shown in B2 in Figure 4). Both options aim to fill the food gap. This will also intensify the pressure on food feed competitions for land and resources to cover food demand (red arrows in Figure 4). If this process generates additional profit, the pressure on resources will increase and will end in higher food demand (not shown).

As a positive effect, vegetable food production and processing will generate a certain amount of agro-industrial by-products that might be used as “human inedible concentrates” for animal nutrition as grain and meal substitutes. The substitution rate will depend on the fibrous, lipid, protein, and energy content of these by-products (e.g., soybean meal vs. whole soybean, soyhulls vs. forages, etc.,). This availability will quantitatively reduce the use of human edible grains in animal feed and, in turn, reduce food–feed competition (the balancing loop B3 and blue arrows in Figure 4). Possible additional beneficial effects on animal productivity were also demonstrated.

When used in animal nutrition, agro-industrial by-products will also provide bioactive compounds that could have beneficial effects for the environment, such as reducing enteric methane and nitrogen excretion (e.g., tannins) or increasing the nutraceutical value of human food from animal sources (e.g., fatty acids, antioxidants, etc.; see balancing loop B4 in Figure 4). The higher the carryover of bioactive compounds from vegetables and by-products to human food, the higher the efficacy of the food supply chain in quantitatively and qualitatively covering and filling the food gap.

## 5. Conclusions

Even if the small ruminant industry is concentrated in few areas, its role could be locally important, mainly in the rural communities of Mediterranean countries [150]. The use of agro-industrial by-products rich in polyphenols in the feeding and nutrition of small dairy ruminants, discounting some conflicting results in the literature, can be considered as a useful strategy to enhance these biomasses by using them in animal feeds. Different productive, metabolic, and managerial aspects often correlated are advantageously improved: better protein utilization, reduction of enteric nitrogen and methane emissions, an improved antioxidant status of the animals, better animal health, improved quality of milk and dairy products, and a reduction of the feed cost and demand for imported feedstuff.

The effective large-scale use of BPRPs in farming systems, such as dairy small ruminant farming (characterized by small farm sizes and territorial rarefaction), is linked to solving concrete problems related to production sources, to the treatment of by-products for their storage, and to the possible inclusion of by-products in commercial feeds. The adoption of low cost and low environmental impact drying systems downstream from the industrial processes is a strategic node that must be resolved to fully insert these important foods into the circular economy.

Looking to the future, the increase in importance of processed foods will make industrial by-products and co-products more available: new technologies for standardization and sanitization will make it increasingly convenient to use these foods for livestock.

## Figures and Tables

**Table 1:** The chemical composition of agro-industrial by-products naturally rich in polyphenols used in dairy small ruminant feeding and nutrition.

Chemical Composition ^1^										
By-Products	DM	OM	NDF	ADF	NFC	CP	Lignin	EE	Ash	Reference
Apple	179	-	107	80	842	4	24	-	-	[18]
Citrus pulp	904	831	194	128	510 *	77	-	49	168	[19]
Citrus pulp	937	-	230	162	604 *	50	38	26	90	[15]
Exhausted myrtle berries	970	-	670	533	292	80	348	110	28	[16]
Exhausted myrtle berries	943	-	648	517	183	78	308	54	37	[17]
Ficus bengalensis	-	852	425	369	279 *	109	-	39	148	[20]
Grape marc	910	938	558	465	-	113	-	89	-	[21]
Grape marc	934	-	527	389	206	111	260	69	87	[17]
Grape pomace	525	940	568	476	-	94	200	52	-	[22]
Grape pomace	890		471	312	-	128	-	-	-	[23]
Grape pomace	439	918	474	440	263	95	-	85	82	[24]
Grape pomace	950	-	376	317	-	119	-	73	89	[25]
Grape pomace	-	866	376	317	-	122	207	64	-	[26]
Grape pulp	-	811	243	193	-	138	75	32	-	[27]
Grape residual flour	934	-	333	217	-	103	-	50	124	[28]
Grape seed	974		539	-	231	93	411	109	27	[29]
Grape seed	-	927	523	454	-	104	353	99	-	[27]
Olive cake	908	-	665	497	-	32.8	308	221	19	[30]
Olive cake	805	901	676	544	-	73	289	54	-	[31]
Olive cake	947	864	584	459	109	79	237	92	136	[32]
Olive cake (exhausted)	950	-	683	531	317	102	367	12	97	[33]
Orange residue (fresh)	219	-	227	171	657 *	60	17	24	32	[34]
Orange peel	266	-	100	76		35	18	17	38	[15]
Pistachio	900	755	259	-	-	153	-	58	-	[35]
Pomegranate (peel)	961	-	208	151	696	36	-	6	54	[36]
Pomegranate pulp	912	-	314	228	-	69	69	26	36	[37]
Pomegranate (seeds)	951	-	680	490	135	154	-	6	24	[36]
Tomato fruit	69	-	260	217	465 *	170	195	28	77	[38]
Tomato pomace	952	952	552	462	109	191	259	100	48	[32]
Tomato pomace	85.1	966	500	340	-	194	-	-	-	[39]
Tomato pomace	94.1	955	554	422	-	217	-	93	-	[40]
Tomato pomace	926	-	616	507	121	157	313	62	44	[17]
Tomato whole plant	177	-	457	356	276 *	74	128	12	181	[38]
Wet tomato pomace	142	962	636	435	-	195	-	-	-	[41]
Winery sediment	312	786	64	43	496	28	-	280	214	[24]

^1^ DM = dry matter, g/kg as fed; OM = organic matter, g/kg DM; NDF = neutral detergent fiber, g/kg DM; ADF = acid detergent fiber, g/kg DM; NFC non fibrous carbohydrates, g/kg DM; CP = crude protein, g/kg DM; Lignin, g/kg DM; EE = eter extract; ash, g/kg DM. * Values were calculated by the authors as follows: NFC (g/kg DM) = 100 − (NDF + CP + ash + EE).

**Table 2:** Processed foods and the relative agro-industrial by-products naturally rich in polyphenols, with the main polyphenols (classes or single compounds) used in small ruminant feeding and nutrition.

Processed Food	By-Product	TP ^1^	TT ^2^	CT ^3^	HT ^4^	Polyphenols	References
*Citrus fruit*	Orange peel	104–223	-	-	-	Gallic acid, ferulic acid, *p*-coumaric, catechins, epicatechins, hesperidin, quercetin, kaempferol	[50]
*Date palm*	Date seeds (pits)	12.7–47.7				Hydroxytyrosol, tyrosol oleuropein, gallic acid, ferulic acid, coumaric acids, *p*-hydroxybenzoic acid, flavonoids	[51,52,53,54]
*Grape*	Grape pomace	14.8–70.5	39.1–105			Anthocyanins, condensed tannins, catechin, epicatechin, gallic acids	[17,55]
	Grape stalk					Flavanols, condensed tannins, flavonols and hydroxycinnamates	[56]
	Grape seeds	3–90				Condensed tannins, catechin, gallic, caffeic, and ferulic acids	[29,57,58]
*Myrtus communis*	Whole exhausted Mirtle berries	47	nd	0.0004	5.7	Hydrolysable tannins, phenolic acids, flavanols, flavonols	[16,17,49]
	Mirtle seeds	39.3	nd	0		phenolic acids, flavanols, flavonols	[49,59]
	Mirtle pericarp	13.7	nd	0.0004	nd	phenolic acids, flavanols, flavonols	[49,59]
*Olive*	Olive cake	4.1–19.4	1.7			Tyrosol, hydroxitirosol, oleuropein, verbacoside, rutin, luteolin, apigenin, quercetin	[32,60,61,62]
	Olive waste water	5.17–8.90 ^5^				Hydroxitirosol, oleuropein, tyrosol, syringing acid, caffeic acid, vanillic acid, verbacoside, catechol, rutin	[60,61,62]
	Olive stones and seeds					Tyrosol, hydroxitirosol, oleuropein, verbacoside (in seeds), nüzhenide in (seeds)	[63]
*Pistachio*	Pistachio hulls	78.5–103	31.6–63.9	8.5–12.0		Gallotannins, flavonoids, anacardic acids.	[40,64,65]
*Pomegranate*	Pomegranate seeds	27.2	16.9	0.8	-	Flavonoids, anthocyanins, hydrolizable tannins	[66]
	Pomegranate peel	48.3	-	-	-	Gallic acid, flavonoids, hydrolizable tannins, condensed tannins, punicalagin	[67,68]
	Pomegranate pulp	95.3	93.4	-	-	Tannins	[69]
*Tomato*	Tomato pomace	6.1–6.4	4.0	0		Naringenin, rutin, quercetin, kaempferol	[17,32,40]

^1^ TP = total phenols, g/kg DM; ^2^ TT = total tannins, g/kg DM, ^3^ CT = condensed tannins, g/kg DM; ^4^ HT = hydrolysable tannins, g/kg DM; ^5^ expressed as g GAE/L.

**Table 3:** The main effects on the rumen parameters of the inclusion of by-products naturally rich in polyphenols (BPRP) in the diets of dairy small ruminants.

BPRP	TP ^1^ in BPRP	TP ^1^ in Diet	Main Effect	Species	Reference
Date palm	-	10.1, 12.6 g/kg DM	Increase pH, propionate and valerate; reduced acetate	goats	[94]
Olive by-product	-	-	Increase nutrient apparent digestibility and metabolizable energy	goat	[127]
Pistachio	-	33.1 g/kg DM	Reduction of ammonia and acetic acid	goat	[107]
Pistachio hull	103 g/kg DM	26.6 g/kg DM	Reduction of ammonia and VFA	goat	[40]
Tomato silage	-	-	Increase nutrient apparent digestibility and metabolizable energy Reduce acetate concentration and (numerically) methane production	goat	[127]
Grape pomace	70.5 g/kg DM	40.7 g/kg DM	Reduction of ammonia, pH, CP digestibility	sheep	[22]
Grape seed	3.0 g/kg DM	0.4 g/kg DM	Increase rumen ammonia, rumenic acid, reduced linoleic and α-linolenic acids	sheep	[29]
Exhausted myrtle berries	50 g/kg DM	2.27 g/kg DM	Reduction of ammonia, VFA, *Butyrivibrio* group	sheep	[123]
Olive oil pomace	-	4.9, 2.7 g/kg DM	Increase α-linolenic and rumenic acids	sheep	[128]
Pistachio by-product	78.5 g/kg DM	42.50 g/kg DM	Decrease total VFA, acetic acid	sheep	[90]
Pistachio hull	78.5 g/kg DM	42.50 g/kg DM	Increase pH, decrease ammonia, total VFA, acetate	sheep	[35]
Pistachio	99.5 g/kg DM	26.4, 35.2g/kg DM	Reduction of ammonia, VFA and acetate	sheep	[92]
Vine leaves	50 g/kg DM	-	Reduce nutrient digestibility	sheep	[108]

^1^ TP = Total polyphenols.

**Table 4:** Effects of the inclusion of by-products naturally rich in polyphenols (BPRP) in the diets of sheep and goats on milk yield and composition.

By-Products	TP ^1^ in by-Products	By-Product in the Diet ^2^	TP ^1^ in Diet g/kg DM	Milk	Fat	Protein	Lactose	Urea	Species	References
lentil straw	2.8 TAE% on DM	300.0	13.20	↓ ns	↓	↑ ns	↑ ns	-	sheep	[32]
atriplex leaves	0.63 TAE% on DM	300.0	5.70	↓ ns	↓ ns	↑ ns	↑	-	sheep	[32]
date palm	-	60.0	9.60	ns	ns	ns	ns	-	goats	[94]
date palm	-	120.0	1.08	ns	ns	ns	ns	-	goats	[94]
date palm	-	180.0	1.26	ns	ns	ns	ns	-	goats	[94]
exhausted myrtle berries	5.30 g GAE/100gDM	22.6	1.20	ns	ns	ns	ns	↓ ns	sheep	[16]
exhausted myrtle berries	5.30 g GAE/100 g DM	44.3	2.35	ns	ns	ns	ns	↓	sheep	[16]
exhausted myrtle berries	40.9 g/kg DM	28.0	1.14	↓	ns	ns	↓	↑ ns	sheep	[17]
grape pomace	14.8 g/kg DM	36.5	0.54	↑	↓	↓	ns	↓ ns	sheep	[17]
grape pomace	42.8 g/kg DM	51.7	2.21	ns	ns	ns	↓	-	sheep	[26]
grape pomace	42.8 g/kg DM	103.2	4.42	ns	ns	ns	↓	-	sheep	[26]
grape residue flour	87.4 mg GAE/g DM	3.4	0.03	ns	↑ ns	ns	ns	-	sheep	[28]
grape residue flour	87.4 mg GAE/g DM	6.7	0.10	ns	↑	ns	ns	-	sheep	[28]
grape seed	0.3g/100 g DM	121.5	0.36	ns	ns	ns	ns	-	sheep	[91]
olive leaves	6.35 TAE% on DM	300.0	22.50	↓	↓ ns	↑ ns	ns	-	sheep	[32]
olive cake	0.41 TAE% on DM	300.0	5.30	↓ ns	ns	ns	↑	-	sheep	[32]
olive silage	-	202.0	-	-	↑	ns	ns	-	goats	[127]
pomegranate seed	-	60.0	-	ns	↑	ns	↑ ns	-	goats	[130]
pomegranate seed	-	120.0	-	ns	↑	ns	↑	-	goats	[130]
pomegranate pulp	95.3 g/kg DM	648.4	61.40	ns	ns	ns	ns	ns	sheep	[37]
RO ^3^ by-product	-	50.0	-	-	ns	ns	ns	-	goats	[131]
RO ^3^ by-product	-	100.0	-	-	ns	ns	↓	-	goats	[131]
tomato pomace	0.64 TAE% on DM	300.0	6.00	↓ ns	↓ ns	↓ ns	↑	-	sheep	[32]
tomato	2.3 g/kg DM	36.2	0.08	ns	↓	↓	ns	↓ ns	sheep	[17]
tomato silage	-	202.0	-	-	↑	ns	ns	-	goats	[127]

↑ = increased; ↓ = decreased; ns = not significant; ↑ ns and ↓ ns = increase and decrease (respectively) tendent to be significant (*p* < 0.10); values were compared to the control (*p* < 0.05). ^1^ TP = total polyphenols. ^2^ expressed as g/kg of DM. ^3^ RO = *Rosmarinus officinalis*.

## References

[1] Environment Action Programme—European Commission. https://ec.europa.eu/environment/action-programme/index.htm.

[2] Scotto A.L. (2012). Impatto Ambientale dei Rifiuti e Degli Sprechi Agroalimentari in Europa e in Italia.

[3] EUROSTAT 2019 (Statistical Office of the European Union). https://ec.europa.eu/eurostat/data/database.

[4] FAOSTAT (Food and Agriculture Organization of the United Nations Statistics Division) (2019). Statistical Database of the Food and Agriculture Organization of the United Nations. http://www.fao.org/faostat/en/?#data/GE.

[5] Federici F., Fava F., Kalogerakis N., Mantzavinos D. (2009). Valorisation of agro-industrial by-products, effluents and waste: concept, opportunities and the case of olive mill wastewaters. J. Chem. Technol. Biotechnol..

[6] Mirzaei-Aghsaghali A., Maheri-Sis N. (2008). Nutritive value of some agro-industrial by-products for ruminants—A review. World J. Zool..

[7] Schieber A., Stintzing F.C., Carle R. (2001). By-products of plant food processing as a source of functional compounds—Recent developments. Trends Food Sci. Technol..

[8] Vasta V., Luciano G. (2011). The effects of dietary consumption of plants secondary compounds on small ruminants’ products quality. Small Rumin. Res..

[9] Min B.R., Barry T.N., Attwood G.T., McNabb W.C. (2003). The effect of condensed tannins on the nutrition and health of ruminants fed fresh temperate forages: A review. Anim. Feed Sci. Technol..

[10] Vasta V., Daghio M., Cappucci A., Buccioni A., Serra A., Viti C., Mele M. (2019). Invited review: Plant polyphenols and rumen microbiota responsible for fatty acid biohydrogenation, fiber digestion, and methane emission: Experimental evidence and methodological approaches. J. Dairy Sci..

[11] Descalzo A.M., Sancho A.M. (2008). A review of natural antioxidants and their effects on oxidative status, odor and quality of fresh beef produced in Argentina. Meat Sci..

[12] Provenza F.D., Kronberg S.L., Gregorini P. (2019). Is Grassfed Meat and Dairy Better for Human and Environmental Health?. Front. Nutr..

[13] Salami S.A., Luciano G., O’Grady M.N., Biondi L., Newbold C.J., Kerry J.P., Priolo A. (2019). Sustainability of feeding plant by-products: A review of the implications for ruminant meat production. Anim. Feed Sci. Technol..

[14] Halmemies-Beauchet-Filleau A., Rinne M., Lamminen M., Mapato C., Ampapon T., Wanapat M., Vanhatalo A. (2018). Review: Alternative and novel feeds for ruminants: Nutritive value, product quality and environmental aspects. Animal.

[15] Castrica M., Rebucci R., Giromini C., Tretola M., Cattaneo D., Baldi A. (2019). Total phenolic content and antioxidant capacity of agri-food waste and by-products. Ital. J. Anim. Sci..

[16] Nudda A., Correddu F., Atzori A.S., Marzano A., Battacone G., Nicolussi P., Bonelli P., Pulina G. (2017). Whole exhausted berries of *Myrtus communis* L. supplied to dairy ewes: Effects on milk production traits and blood metabolites. Small Rumin. Res..

[17] Nudda A., Buffa G., Atzori A.S., Cappai M.G., Caboni P., Fais G., Pulina G. (2019). Small amounts of agro-industrial byproducts in dairy ewes diets affects milk production traits and hematological parameters. Anim. Feed Sci. Technol..

[18] Rodrigues M.A.M., Guedes C.M., Rodrigues A., Cone J.W., van Gelder A.H., Ferreira L.M.M. (2008). Evaluation of the nutritive value of apple pulp mixed with different amounts of wheat straw. Livest. Res. Rural Dev..

[19] Fegeros K., Zervas G., Stamouli S., Apostolaki E. (1995). Nutritive Value of Dried Citrus Pulp and Its Effect on Milk Yield and Milk Composition of Lactating Ewes. J. Dairy Sci..

[20] Dey A., De P.S. (2014). Influence of Condensed Tannins from Ficus bengalensis Leaves on Feed Utilization, Milk Production and Antioxidant Status of Crossbred Cows. Asian-Australas. J. Anim. Sci..

[21] Tsiplakou E., Zervas G. (2008). The effect of dietary inclusion of olive tree leaves and grape marc on the content of conjugated linoleic acid and vaccenic acid in the milk of dairy sheep and goats. J. Dairy Res..

[22] Abarghuei M.J., Rouzbehan Y., Alipour D. (2010). The influence of the grape pomace on the ruminal parameters of sheep. Livest. Sci..

[23] Bahrami Y., Foroozandeh A.D., Zamani F., Modarresi M., Eghbal-Saeid S., Chekani-Azar S. (2010). Effect of diet with varying levels of dried grape pomace on dry matter digestibility and growth performance of male lambs. J. Anim. Plant Sci..

[24] Ishida K., Kishi Y., Oishi K., Hirooka H., Kumagai H. (2015). Effects of feeding polyphenol-rich winery wastes on digestibility, nitrogen utilization, ruminal fermentation, antioxidant status and oxidative stress in wethers. Anim. Sci. J..

[25] Guerra-Rivas C., Vieira C., Rubio B., Martínez B., Gallardo B., Mantecón A.R., Lavín P., Manso T. (2016). Effects of grape pomace in growing lamb diets compared with vitamin E and grape seed extract on meat shelf life. Meat Sci..

[26] Manso T., Gallardo B., Salvá A., Guerra-Rivas C., Mantecón A.R., Lavín P., de la Fuente M.A. (2016). Influence of dietary grape pomace combined with linseed oil on fatty acid profile and milk composition. J. Dairy Sci..

[27] Guerra-Rivas C., Gallardo B., Mantecón Á.R., Álamo-Sanza M., del Manso T. (2017). Evaluation of grape pomace from red wine by-product as feed for sheep. J. Sci. Food Agric..

[28] Alba D.F., Campigotto G., Cazarotto C.J., dos Santos D.S., Gebert R.R., Reis J.H., Souza C.F., Baldissera M.D., Gindri A.L., Kempka A.P. (2019). Use of grape residue flour in lactating dairy sheep in heat stress: Effects on health, milk production and quality. J. Therm. Biol..

[29] Correddu F., Nudda A., Battacone G., Boe R., Francesconi A.H.D., Pulina G. (2015). Effects of grape seed supplementation, alone or associated with linseed, on ruminal metabolism in Sarda dairy sheep. Anim. Feed Sci. Technol..

[30] Chiofalo B., Liotta L., Zumbo A., Chiofalo V. (2004). Administration of olive cake for ewe feeding: Effect on milk yield and composition. Small Rumin. Res..

[31] Molina-Alcaide E., Yáñez-Ruiz D.R. (2008). Potential use of olive by-products in ruminant feeding: A review. Anim. Feed Sci. Technol..

[32] Abbeddou S., Rischkowsky B., Richter E.K., Hess H.D., Kreuzer M. (2011). Modification of milk fatty acid composition by feeding forages and agro-industrial byproducts from dry areas to Awassi sheep. J. Dairy Sci..

[33] Tufarelli V., Introna M., Cazzato E., Mazzei D., Laudadio V. (2013). Suitability of partly destoned exhausted olive cake as by-product feed ingredient for lamb production. J. Anim. Sci..

[34] Villanueva Z., Ibarra M.A., Briones F., Escamilla O.S. (2013). Productive performance of hair lambs fed fresh orange (*Citrus sinensis*) residues substituting sorghum (*Sorghum vulgare*) grains. Cuban J. Agric. Sci..

[35] Ghasemi S., Naserian A.A., Valizadeh R., Tahmasebi A.M., Vakili A.R., Behgar M., Ghovvati S. (2012). Inclusion of pistachio hulls as a replacement for alfalfa hay in the diet of sheep causes a shift in the rumen cellulolytic bacterial population. Small Rumin. Res..

[36] Mirzaei-Aghsaghali A., Maheri-Sis N., Mansouri H., Razeghi M.E., Mirza-Aghazadeh A., Cheraghi H., Aghajanzadeh-Golshani A. (2011). Evaluating potential nutritive value of pomegranate processing by-products for ruminants using in vitro gas production technique. ARPN J. Agric. Biol. Sci..

[37] Valenti B., Luciano G., Morbidini L., Rossetti U., Codini M., Avondo M., Priolo A., Bella M., Natalello A., Pauselli M. (2019). Dietary pomegranate pulp: Effect on ewe milk quality during late lactation. Animals.

[38] Ventura M.R., Pieltain M.C., Castanon J.I.R. (2009). Evaluation of tomato crop by-products as feed for goats. Anim. Feed Sci. Technol..

[39] Shdaifat M.M., Al-Barakah F.S., Kanan A.Q., Obeidat B.S. (2013). The effect of feeding agricultural by-products on performance of lactating Awassi ewes. Small Rumin. Res..

[40] Razzaghi A., Naserian A.A., Valizadeh R., Ebrahimi S.H., Khorrami B., Malekkhahi M., Khiaosa-ard R. (2015). Pomegranate seed pulp, pistachio hulls, and tomato pomace as replacement of wheat bran increased milk conjugated linoleic acid concentrations without adverse effects on ruminal fermentation and performance of Saanen dairy goats. Anim. Feed Sci. Technol..

[41] Denek N., Can A. (2006). Feeding value of wet tomato pomace ensiled with wheat straw and wheat grain for Awassi sheep. Small Rumin. Res..

[42] Naczk M., Shahidi F. (2004). Extraction and analysis of phenolics in food. J. Chromatogr. A.

[43] Quideau S., Deffieux D., Douat-Casassus C., Pouységu L. (2011). Plant polyphenols: chemical properties, biological activities, and synthesis. Angew. Chem. Int. Ed..

[44] Bravo L. (1998). Polyphenols: Chemistry, dietary sources, metabolism, and nutritional significance. Nutr. Rev..

[45] Frutos P., Hervás G., Giráldez F.J., Mantecón A.R. (2004). Review. Tannins and ruminant nutrition. Span. J. Agric. Res..

[46] Montoro P., Tuberoso C.I.G., Piacente S., Perrone A., De Feo V., Cabras P., Pizza C. (2006). Stability and antioxidant activity of polyphenols in extracts of Myrtus communis L. berries used for the preparation of myrtle liqueur. J. Pharm. Biomed. Anal..

[47] Maldini M., Chessa M., Petretto G.L., Montoro P., Rourke J.P., Foddai M., Nicoletti M., Pintore G. (2016). Profiling and simultaneous quantitative determination of anthocyanins in wild *Myrtus communis* L. Berries from different geographical areas in Sardinia and their comparative evaluation. Phytochem. Anal..

[48] Tuberoso C.I.G., Rosa A., Bifulco E., Melis M.P., Atzeri A., Pirisi F.M., Dessì M.A. (2010). Chemical composition and antioxidant activities of *Myrtus communis* L. berries extracts. Food Chem..

[49] Correddu F., Maldini M., Addis R., Petretto G.L., Palomba M., Battacone G., Pulina G., Nudda A., Pintore G. (2019). Myrtus communis liquor byproduct as a source of bioactive compounds. Foods.

[50] Ozturk B., Parkinson C., Gonzalez-Miquel M. (2018). Extraction of polyphenolic antioxidants from orange peel waste using deep eutectic solvents. Sep. Purif. Technol..

[51] Besbes S., Blecker C., Deroanne C., Bahloul N., Lognay G., Drira N.-E., Attia H. (2004). Date seed oil: Phenolic, tocopherol and sterol profiles. J. Food Lipids.

[52] Al-Farsi M.A., Lee C.Y. (2008). Optimization of phenolics and dietary fibre extraction from date seeds. Food Chem..

[53] Shams Ardekani M.R., Khanavi M., Hajimahmoodi M., Jahangiri M., Hadjiakhoondi A. (2010). Comparison of Antioxidant Activity and Total Phenol Contents of some Date Seed Varieties from Iran. Iran. J. Pharm. Res..

[54] Platat C., M Habib H., AL Maqbali F.D., Jaber N.N., Ibrahim W.H. (2014). Identification of date seeds varieties patterns to optimize nutritional benefits of date seeds. Nutr. Food Sci..

[55] Bonilla F., Mayen M., Merida J., Medina M. (1999). Extraction of phenolic compounds from red grape marc for use as food lipid antioxidants. Food Chem..

[56] Alonso Á.M., Guillén D.A., Barroso C.G., Puertas B., García A. (2002). Determination of Antioxidant Activity of Wine Byproducts and Its Correlation with Polyphenolic Content. J. Agric. Food Chem..

[57] Spanghero M., Salem A.Z.M., Robinson P.H. (2009). Chemical composition, including secondary metabolites, and rumen fermentability of seeds and pulp of Californian (USA) and Italian grape pomaces. Anim. Feed Sci. Technol..

[58] Lutterodt H., Slavin M., Whent M., Turner E., Yu L. (2011). Fatty acid composition, oxidative stability, antioxidant and antiproliferative properties of selected cold-pressed grape seed oils and flours. Food Chem..

[59] Correddu F., Nudda A., Pulina G. Effect of Myrtus communis liquor by-product on milk FA profile of Sarda dairy sheep.

[60] Obied H.K., Bedgood D.R., Prenzler P.D., Robards K. (2007). Bioscreening of Australian olive mill waste extracts: Biophenol content, antioxidant, antimicrobial and molluscicidal activities. Food Chem. Toxicol..

[61] Suárez M., Romero M.-P., Ramo T., Macià A., Motilva M.-J. (2009). Methods for Preparing Phenolic Extracts from Olive Cake for Potential Application as Food Antioxidants. J. Agric. Food Chem..

[62] Leouifoudi I., Harnafi H., Zyad A. (2015). Olive mill waste extracts: Polyphenols content, antioxidant, and antimicrobial activities. Adv. Pharmacol. Sci..

[63] Ryan D., Prenzler P.D., Lavee S., Antolovich M., Robards K. (2003). Quantitative Changes in Phenolic Content during Physiological Development of the Olive (*Olea europaea*) Cultivar Hardy’s Mammoth. J. Agric. Food Chem..

[64] Erşan S., Güçlü Üstündağ Ö., Carle R., Schweiggert R.M. (2016). Identification of Phenolic Compounds in Red and Green Pistachio (*Pistacia vera* L.) Hulls (Exo- and Mesocarp) by HPLC-DAD-ESI-(HR)-MSn. J. Agric. Food Chem..

[65] Grace M.H., Esposito D., Timmers M.A., Xiong J., Yousef G., Komarnytsky S., Lila M.A. (2016). Chemical composition, antioxidant and anti-inflammatory properties of pistachio hull extracts. Food Chem..

[66] Elfalleh W. (2012). Total phenolic contents and antioxidant activities of pomegranate peel, seed, leaf and flower. J. Med. Plants Res..

[67] Ambigaipalan P., de Camargo A.C., Shahidi F. (2016). Phenolic Compounds of Pomegranate Byproducts (Outer Skin, Mesocarp, Divider Membrane) and Their Antioxidant Activities. J. Agric. Food Chem..

[68] Gullon B., Pintado M.E., Pérez-Álvarez J.A., Viuda-Martos M. (2016). Assessment of polyphenolic profile and antibacterial activity of pomegranate peel (*Punica granatum*) flour obtained from co-product of juice extraction. Food Control.

[69] Abarghuei M.J., Rouzbehan Y., Salem A.Z.M., Zamiri M.J. (2014). Nitrogen balance, blood metabolites and milk fatty acid composition of dairy cows fed pomegranate-peel extract. Livest. Sci..

[70] Rice-evans C.A., Miller N.J., Bolwell P.G., Bramley P.M., Pridham J.B. (1995). The relative antioxidant activities of plant-derived polyphenolic Flavonoids. Free Radic. Res..

[71] Schroeter H., Heiss C., Balzer J., Kleinbongard P., Keen C.L., Hollenberg N.K., Sies H., Kwik-Uribe C., Schmitz H.H., Kelm M. (2006). Epicatechin mediates beneficial effects of flavanol-rich cocoa on vascular function in humans. Proc. Natl. Acad. Sci. USA.

[72] Perez-Vizcaino F., Duarte J., Jimenez R., Santos-Buelga C., Osuna A. (2009). Antihypertensive effects of the flavonoid quercetin. Pharmacol. Rep..

[73] Kubena K.S., McMurray D.N. (1996). Nutrition and the Immune System: A Review of Nutrient–Nutrient Interactions. J. Am. Diet. Assoc..

[74] Bahadoran Z., Mirmiran P., Azizi F. (2013). Dietary polyphenols as potential nutraceuticals in management of diabetes: A review. J. Diabetes Metab. Disord..

[75] Stoner G.D., Mukhtar H. (1995). Polyphenols as cancer chemopreventive agents. J. Cell. Biochem..

[76] Forester S.C., Choy Y.Y., Waterhouse A.L., Oteiza P.I. (2014). The anthocyanin metabolites gallic acid, 3-O-methylgallic acid, and 2,4,6-trihydroxybenzaldehyde decrease human colon cancer cell viability by regulating pro-oncogenic signals. Mol. Carcinog..

[77] Koeberle A., Werz O. (2014). Multi-target approach for natural products in inflammation. Drug Discov. Today.

[78] Makkar H.P.S. (2003). Effects and fate of tannins in ruminant animals, adaptation to tannins, and strategies to overcome detrimental effects of feeding tannin-rich feeds. Small Rumin. Res..

[79] Wang Y., Waghorn G.C., McNabb W.C., Barry T.N., Hedley M.J., Shelton I.D. (1996). Effect of condensed tannins in Lotus corniculatus upon the digestion of methionine and cysteine in the small intestine of sheep. J. Agric. Sci..

[80] Toral P.G., Hervás G., Bichi E., Belenguer Á., Frutos P. (2011). Tannins as feed additives to modulate ruminal biohydrogenation: Effects on animal performance, milk fatty acid composition and ruminal fermentation in dairy ewes fed a diet containing sunflower oil. Anim. Feed Sci. Technol..

[81] Toral P.G., Monahan F.J., Hervás G., Frutos P., Moloney A.P. (2018). Review: Modulating ruminal lipid metabolism to improve the fatty acid composition of meat and milk. Challenges and opportunities. Animal.

[82] Athanasiadou S., Kyriazakis I., Jackson F., Coop R.L. (2001). Direct anthelmintic effects of condensed tannins towards different gastrointestinal nematodes of sheep: In vitro and in vivo studies. Vet. Parasitol..

[83] Athanasiadou S., Kyriazakis I., Jackson F. (2006). Can plant secondary metabolites have a role in controlling gastrointestinal nematode parasitism in small ruminants?. BSAP Occas. Publ..

[84] Pathak A.K. (2013). Potential of using condensed tannins to control gastrointestinal nematodes and improve small ruminant performance. Int. J. Mol. Vet. Res..

[85] Gessner D.K., Ringseis R., Eder K. (2017). Potential of plant polyphenols to combat oxidative stress and inflammatory processes in farm animals. J. Anim. Physiol. Anim. Nutr..

[86] Liu H.W., Zhou D.W., Li K. (2013). Effects of chestnut tannins on performance and antioxidative status of transition dairy cows. J. Dairy Sci..

[87] López-Andrés P., Luciano G., Vasta V., Gibson T.M., Biondi L., Priolo A., Mueller-Harvey I. (2013). Dietary quebracho tannins are not absorbed, but increase the antioxidant capacity of liver and plasma in sheep. Br. J. Nutr..

[88] Alcaide E.M., Ruiz D.Y., Moumen A., Garcıa A.M. (2003). Ruminal degradability and in vitro intestinal digestibility of sunflower meal and in vitro digestibility of olive by-products supplemented with urea or sunflower meal: comparison between goats and sheep. Anim. Feed Sci. Technol..

[89] Yáñez Ruiz D.R., Moumen A., Martin Garcia A.I., Molina Alcaide E. (2004). Ruminal fermentation and degradation patterns, protozoa population, and urinary purine derivatives excretion in goats and wethers fed diets based on two-stage olive cake: Effect of PEG supply. J. Anim. Sci..

[90] Ghasemi S., Naserian A.A., Valizadeh R., Tahmasebi A.M., Vakili A.R., Behgar M. (2012). Effects of pistachio by-product in replacement of lucerne hay on microbial protein synthesis and fermentative parameters in the rumen of sheep. Anim. Prod. Sci..

[91] Nudda A., Correddu F., Marzano A., Battacone G., Nicolussi P., Bonelli P., Pulina G. (2015). Effects of diets containing grape seed, linseed, or both on milk production traits, liver and kidney activities, and immunity of lactating dairy ewes. J. Dairy Sci..

[92] Ghaffari M.H., Tahmasbi A.M., Khorvash M., Naserian A.A., Ghaffari A.H., Valizadeh H. (2014). Effects of pistachio by-products in replacement of alfalfa hay on populations of rumen bacteria involved in biohydrogenation and fermentative parameters in the rumen of sheep. J. Anim. Physiol. Anim. Nutr..

[93] Sedighi-Vesagh R., Naserian A.A., Ghaffari M.H., Petit H.V. (2015). Effects of pistachio by-products on digestibility, milk production, milk fatty acid profile and blood metabolites in Saanen dairy goats. J. Anim. Physiol. Anim. Nutr..

[94] Sharifi M., Bashtani M., Naserian A.A., Farhangfar H. (2015). The effect of feeding low quality date palm (*Phoenix dactylifera* L.) on the performance, antioxidant status and ruminal fermentation of mid-lactating Saanen dairy goats. Small Rumin. Res..

[95] Hofmann R.R. (1989). Evolutionary steps of ecophysiological adaptation and diversification of ruminants: A comparative view of their digestive system. Oecologia.

[96] Alonso-Díaz M.A., Torres-Acosta J.F.J., Sandoval-Castro C.A., Capetillo-Leal C.M. (2012). Amino acid profile of the protein from whole saliva of goats and sheep and its interaction with tannic acid and tannins extracted from the fodder of tropical plants. Small Rumin. Res..

[97] Domingue B.M.F., Dellow D.W., Barry T.N. (1991). The efficiency of chewing during eating and ruminating in goats and sheep. Br. J. Nutr..

[98] Lamy E., da Costa G., e Silva F.C., Potes J., Coelho A.V., Baptista E.S. (2008). Comparison of Electrophoretic Protein Profiles from Sheep and Goat Parotid Saliva. J. Chem. Ecol..

[99] Lamy E., Rawel H., Schweigert F.J., Capela e Silva F., Ferreira A., Costa A.R., Antunes C., Almeida A.M., Coelho A.V., Sales-Baptista E. (2011). The Effect of Tannins on Mediterranean Ruminant Ingestive Behavior: The Role of the Oral Cavity. Molecules.

[100] Leparmarai P.T., Sinz S., Kunz C., Liesegang A., Ortmann S., Kreuzer M., Marquardt S. (2019). Transfer of total phenols from a grapeseed-supplemented diet to dairy sheep and goat milk, and effects on performance and milk quality. J. Anim. Sci..

[101] Austin P.J., Suchar L.A., Robbins C.T., Hagerman A.E. (1989). Tannin-binding proteins in saliva of deer and their absence in saliva of sheep and cattle. J. Chem. Ecol..

[102] Mancilla-Leytón J.M., Vicente A.M., Delgado-Pertíñez M. (2013). Summer diet selection of dairy goats grazing in a Mediterranean shrubland and the quality of secreted fat. Small Rumin. Res..

[103] Silanikove N. (1997). Why goats raised on harsh environment perform better than other domesticated animals. Options Mediterr..

[104] McMahon L.R., McAllister T.A., Berg B.P., Majak W., Acharya S.N., Popp J.D., Coulman B.E., Wang Y., Cheng K.J. (2000). A review of the effects of forage condensed tannins on ruminal fermentation and bloat in grazing cattle. Can. J. Plant Sci..

[105] Schreurs N.M., Tavendale M.H., Lane G.A., Barry T.N., Lopez-Villalobos N., McNabb W.C. (2007). Effect of different condensed tannin-containing forages, forage maturity and nitrogen fertiliser application on the formation of indole and skatole in in vitro rumen fermentations. J. Sci. Food Agric..

[106] Cabiddu A., Salis L., Tweed J.K., Molle G., Decandia M., Lee M.R. (2010). The influence of plant polyphenols on lipolysis and biohydrogenation in dried forages at different phenological stages: in vitro study. J. Sci. Food Agric..

[107] Ghaffari M.H., Tahmasbi A.M., Khorvash M., Naserian A.A., Vakili A.R. (2014). Effects of pistachio by-products in replacement of alfalfa hay on ruminal fermentation, blood metabolites, and milk fatty acid composition in Saanen dairy goats fed a diet containing fish oil. J. Appl. Anim. Res..

[108] Romero M.J., Madrid J., Hernández F., Cerón J.J. (2000). Digestibility and voluntary intake of vine leaves (*Vitis vinifera* L.) by sheep. Small Rumin. Res..

[109] García A.M., Moumen A., Ruiz D.Y., Alcaide E.M. (2003). Chemical composition and nutrients availability for goats and sheep of two-stage olive cake and olive leaves. Anim. Feed Sci. Technol..

[110] Awawdeh M.S. (2011). Alternative feedstuffs and their effects on performance of Awassi sheep: A review. Trop. Anim. Health Prod..

[111] Molina Alcaide E., Yáñez Ruiz D., Moumen A., Martín García I. (2003). Chemical composition and nitrogen availability for goats and sheep of some olive by-products. Small Rumin. Res..

[112] Yáñez-Ruiz D.R., Molina-Alcaide E. (2007). A comparative study of the effect of two-stage olive cake added to alfalfa on digestion and nitrogen losses in sheep and goats. Animal.

[113] Perez-Maldonado R.A., Norton B.W. (1996). Digestion of 14 C-labelled condensed tannins from Desmodium intortum in sheep and goats. Br. J. Nutr..

[114] Brooker J.D., O’donovan L.A., Skene I., Clarke K., Blackall L., Muslera P. (1994). Streptococcus caprinus sp. nov., a tannin-resistant ruminal bacterium from feral goats. Lett. Appl. Microbiol..

[115] Paraskevakis N. (2015). Effects of dietary dried Greek Oregano (*Origanum vulgare* ssp. hirtum) supplementation on blood and milk enzymatic antioxidant indices, on milk total antioxidant capacity and on productivity in goats. Anim. Feed Sci. Technol..

[116] Moñino I., Martínez C., Sotomayor J.A., Lafuente A., Jordán M.J. (2008). Polyphenolic Transmission to Segureño Lamb Meat from Ewes’ Diet Supplemented with the Distillate from Rosemary (*Rosmarinus officinalis*) Leaves. J. Agric. Food Chem..

[117] Jordán M.J., Moñino M.I., Martínez C., Lafuente A., Sotomayor J.A. (2010). Introduction of Distillate Rosemary Leaves into the Diet of the Murciano-Granadina Goat: Transfer of Polyphenolic Compounds to Goats’ Milk and the Plasma of Suckling Goat Kids. J. Agric. Food Chem..

[118] Halliwell B., Rafter J., Jenner A. (2005). Health promotion by flavonoids, tocopherols, tocotrienols, and other phenols: Direct or indirect effects? Antioxidant or not?. Am. J. Clin. Nutr..

[119] Kerem Z., Chetrit D., Shoseyov O., Regev-Shoshani G. (2006). Protection of Lipids from Oxidation by Epicatechin, trans-Resveratrol, and Gallic and Caffeic Acids in Intestinal Model Systems. J. Agric. Food Chem..

[120] Torres-Acosta J.F.J., Hoste H. (2008). Alternative or improved methods to limit gastro-intestinal parasitism in grazing sheep and goats. Small Rumin. Res..

[121] Borges D.G.L., Borges F.D.A. (2016). Plants and their medicinal potential for controlling gastrointestinal nematodes in ruminants. Nematoda.

[122] Windisch W., Schedle K., Plitzner C., Kroismayr A. (2008). Use of phytogenic products as feed additives for swine and poultry. J. Anim. Sci..

[123] Correddu F., Fancello F., Chessa L., Atzori A.S., Pulina G., Nudda A. (2019). Effects of supplementation with exhausted myrtle berries on rumen function of dairy sheep. Small Rumin. Res..

[124] Tavendale M.H., Meagher L.P., Pacheco D., Walker N., Attwood G.T., Sivakumaran S. (2005). Methane production from in vitro rumen incubations with Lotus pedunculatus and Medicago sativa, and effects of extractable condensed tannin fractions on methanogenesis. Anim. Feed Sci. Technol..

[125] Goel G., Makkar H.P.S. (2012). Methane mitigation from ruminants using tannins and saponins. Trop. Anim. Health Prod..

[126] Bhatta R., Uyeno Y., Tajima K., Takenaka A., Yabumoto Y., Nonaka I., Enishi O., Kurihara M. (2009). Difference in the nature of tannins on in vitro ruminal methane and volatile fatty acid production and on methanogenic archaea and protozoal populations. J. Dairy Sci..

[127] Arco-Pérez A., Ramos-Morales E., Yáñez-Ruiz D.R., Abecia L., Martín-García A.I. (2017). Nutritive evaluation and milk quality of including of tomato or olive by-products silages with sunflower oil in the diet of dairy goats. Anim. Feed Sci. Technol..

[128] Mannelli F., Cappucci A., Pini F., Pastorelli R., Decorosi F., Giovannetti L., Mele M., Minieri S., Conte G., Pauselli M. (2018). Effect of different types of olive oil pomace dietary supplementation on the rumen microbial community profile in Comisana ewes. Sci. Rep..

[129] Marcos C.N., de Evan T., Molina-Alcaide E., Carro M.D. (2019). Nutritive Value of Tomato Pomace for Ruminants and Its Influence on In Vitro Methane Production. Animals.

[130] Modaresi J., Fathi Nasri M.H., Rashidi L., Dayani O., Kebreab E. (2011). Short communication: Effects of supplementation with pomegranate seed pulp on concentrations of conjugated linoleic acid and punicic acid in goat milk. J. Dairy Sci..

[131] Boutoial K., Ferrandini E., Rovira S., García V., López M.B. (2013). Effect of feeding goats with rosemary (*Rosmarinus officinalis* spp.) by-product on milk and cheese properties. Small Rumin. Res..

[132] Abbeddou S., Rischkowsky B., Hilali M.E.-D., Haylani M., Hess H.D., Kreuzer M. (2015). Supplementing diets of Awassi ewes with olive cake and tomato pomace: on-farm recovery of effects on yield, composition and fatty acid profile of the milk. Trop. Anim. Health Prod..

[133] Dehghan M., Mente A., Zhang X., Swaminathan S., Li W., Mohan V., Iqbal R., Kumar R., Wentzel-Viljoen E., Rosengren A. (2017). Associations of fats and carbohydrate intake with cardiovascular disease and mortality in 18 countries from five continents (PURE): A prospective cohort study. Lancet.

[134] Bhattacharya A., Banu J., Rahman M., Causey J., Fernandes G. (2006). Biological effects of conjugated linoleic acids in health and disease. J. Nutr. Biochem..

[135] Sofi F., Buccioni A., Cesari F., Gori A.M., Minieri S., Mannini L., Casini A., Gensini G.F., Abbate R., Antongiovanni M. (2010). Effects of a dairy product (pecorino cheese) naturally rich in cis-9, trans-11 conjugated linoleic acid on lipid, inflammatory and haemorheological variables: A dietary intervention study. Nutr. Metab. Cardiovasc. Dis..

[136] Pintus S., Murru E., Carta G., Cordeddu L., Batetta B., Accossu S., Pistis D., Uda S., Ghiani M.E., Mele M. (2013). Sheep cheese naturally enriched in α-linolenic, conjugated linoleic and vaccenic acids improves the lipid profile and reduces anandamide in the plasma of hypercholesterolaemic subjects. Br. J. Nutr..

[137] Jenkins T.C., Wallace R.J., Moate P.J., Mosley E.E. (2008). Board-invited review: Recent advances in biohydrogenation of unsaturated fatty acids within the rumen microbial ecosystem. J. Anim. Sci..

[138] Vasta V., Makkar H.P.S., Mele M., Priolo A. (2008). Ruminal biohydrogenation as affected by tannins in vitro. Br. J. Nutr..

[139] Khiaosa-Ard R., Bryner S.F., Scheeder M.R.L., Wettstein H.-R., Leiber F., Kreuzer M., Soliva C.R. (2009). Evidence for the inhibition of the terminal step of ruminal α-linolenic acid biohydrogenation by condensed tannins. J. Dairy Sci..

[140] Correddu F., Gaspa G., Pulina G., Nudda A. (2016). Grape seed and linseed, alone and in combination, enhance unsaturated fatty acids in the milk of Sarda dairy sheep. J. Dairy Sci..

[141] Moate P.J., Williams S.R.O., Torok V.A., Hannah M.C., Ribaux B.E., Tavendale M.H., Eckard R.J., Jacobs J.L., Auldist M.J., Wales W.J. (2014). Grape marc reduces methane emissions when fed to dairy cows. J. Dairy Sci..

[142] Bodas R., Manso T., Mantecón Á.R., Juárez M., De la Fuente M.Á., Gómez-Cortés P. (2010). Comparison of the Fatty Acid Profiles in Cheeses from Ewes Fed Diets Supplemented with Different Plant Oils. J. Agric. Food Chem..

[143] Kliem K.E., Shingfield K.J. (2016). Manipulation of milk fatty acid composition in lactating cows: Opportunities and challenges. Eur. J. Lipid Sci. Technol..

[144] Nudda A., Battacone G., Boaventura Neto O., Cannas A., Francesconi A.H.D., Atzori A.S., Pulina G. (2014). Feeding strategies to design the fatty acid profile of sheep milk and cheese. Revista Brasileira de Zootecnia.

[145] Correddu F., Nudda A., Manca M.G., Pulina G., Dalsgaard T.K. (2015). Light-Induced Lipid Oxidation in Sheep Milk: Effects of Dietary Grape Seed and Linseed, Alone or in Combination, on Milk Oxidative Stability. J. Agric. Food Chem..

[146] Armendáriz V., Armenia S., Atzori A.S. (2016). Systemic Analysis of Food Supply and Distribution Systems in City-Region Systems—An Examination of FAO’s Policy Guidelines towards Sustainable Agri-Food Systems. Agriculture.

[147] Turner B.L., Menendez H.M., Gates R., Tedeschi L.O., Atzori A.S. (2016). System Dynamics Modeling for Agricultural and Natural Resource Management Issues: Review of Some Past Cases and Forecasting Future Roles. Resources.

[148] Molina Benavides R.A., Sánchez Guerrero H., Campos Gaona R., Atzori A.S., Morales J.D., Molina Benavides R.A., Sánchez Guerrero H., Campos Gaona R., Atzori A.S., Morales J.D. (2017). Dynamic estimation of greenhouse gas emissions from bovine livestock of Valle del Cauca, Colombia. Acta Agronómica.

[149] Sterman J. Business Dynamics: Systems Thinking and Modeling for a Complex World 2000. https://dk.um.si/IzpisGradiva.php?id=28601.

[150] Pulina G., Milán M.J., Lavín M.P., Theodoridis A., Morin E., Capote J., Thomas D.L., Francesconi A.H.D., Caja G. (2018). Invited review: Current production trends, farm structures, and economics of the dairy sheep and goat sectors. J. Dairy Sci..

